# Ethnic disparity in metabolic syndrome and related obesity and health behavior: a community study in Taiwan

**DOI:** 10.1186/s13098-021-00751-3

**Published:** 2021-11-17

**Authors:** Chih-Ying Lin, Hui-Jung Hung, Chi-Jung Chung, Chia-Ti Huang, Trong-Neng Wu, Chiu-Ying Chen

**Affiliations:** 1 Department of Public Health, China Medical University, No. 100, Sec. 1, Jingmao Rd., Beitun Dist., Taichung, 40604 Taiwan; 2 Department of Nursing and Graduate Institute of Nursing, Asia University, No. 500, Lioufeng Rd., Wufeng, Taichung, 41354 Taiwan; 3 Medical Affairs Section, Public Health Bureau, Taitung County, No. 336, Bo’ai Rd., Taitung City, Taitung County 95043 Taiwan; 4 Department of Healthcare Administration, Asia University, No. 500, Lioufeng Rd., Wufeng, Taichung, 41354 Taiwan; 5 Graduate Institute of Clinical Medical Science, College of Medicine, China Medical University, No.91, Hsueh-Shih Rd., Taichung, 40402 Taiwan; 6 Department of Senior Citizen Service Management, College of Health, National Taichung University of Science and Technology, No. 193, Sec. 1, Sanmin Rd., Taichung, 40401 Taiwan

**Keywords:** Ethnic disparity, Metabolic syndrome, Obesity, Health behavior, Biological factor

## Abstract

**Background:**

As studies on ethnic disparities in metabolic syndrome and its risk factors in Taiwan are still rare, the aims of this study were: (1) to detect the differences in the rates of metabolic syndrome, obesity and health behaviors between two ethnic groups (indigenous Tsou and nonindigenous Han) living in the same area and with similar age and sex distributions; (2) to examine whether ethnicity per se plays a significant role in the occurrence of metabolic syndrome, while taking other risk factors including sociodemographic characteristics, obesity and health behaviors into consideration.

**Methods:**

This is a cross-sectional study using data from a community survey conducted in Chiayi County in southwestern Taiwan. A frequency matching strategy by age and sex with a ratio of 1 (Tsou) to 3 (Han) was applied to select a comparable sample between both ethnic groups (667 Tsou and 2001 Han) from among the survey participants. Furthermore, participants with cardiometabolic diseases diagnosed before the surveyed day were excluded to avoid confounding any associated risk factors for developing metabolic syndrome (MS). A final analytic sample of 1482 remained. The used information included sociodemographic characteristics, medical histories, health behaviors, and the concentrations of triglycerides, cholesterol, and glucose.

**Results:**

Indigenous Tsou had significantly higher rates of metabolic syndromes, obesity and unhealthy behaviors than their Han counterparts (MS: 54.0% vs. 29.1%, obesity: 54.0% vs. 23.2%, drinking alcohol: 17.5% vs. 13.6%, and higher intake of fried food: 6.4% vs. 4.4%), even though they were similar in age and sex distributions. The significant risk factors for subsequently developing MS included being indigenous Tsou (adjusted POR = 2.62, P < 0.001), older, single, and obese. Stratified analyses on the risk factors for developing MS by health behaviors and by obese problems also indicated increased risks of being indigenous Tsou.

**Conclusions:**

There existed ethnic differences in the rates of metabolic syndrome, obesity, and health behaviors. Ethnicity per se did play a significant role in developing MS; in particular indigenous Tsou people had increased risks, suggesting possible biological reasons rooted in their origins that need further exploration. In addition, unhealthy behaviors may potentially have an indirect effect on developing MS via their effect on obesity.

## Background

In the past two decades, metabolic syndrome (MS) has drawn public attention to the prevention of cardiovascular diseases (CVDs). MS associated with insulin resistance is a clustering of abdominal obesity, hyperglycemia, dyslipidemia, and elevated blood pressure [1]. It usually begins with insulin resistance in early life and proceeds to partial metabolic syndrome and prediabetes before progressing to type 2 diabetes mellitus and cardiovascular disease, which are together referred to as cardiometabolic diseases [2].

According to the definition by the National Cholesterol Education Programme Adult Treatment Panel III (NCEP-ATP III), MS should include three or more of the following five characteristics: abdominal obesity (waist circumference > 102 cm in men and > 88 cm in women), triglycerides ≥ 150 mg/dl, HDL cholesterol < 40 mg/dl in men and < 50 mg/dl in women, blood pressure ≥ 130 mmHg for systolic or ≥ 85 for diastolic, and fasting glucose ≥ 100 mg/dl [3].

Metabolic syndrome, age, male sex, health behaviors, and other unknown factors such as genetic and some biological factors that might difficultly be assessed in clinical practice, are taken as a global cardiometabolic risk (CMR) warning for developing CVD and diabetes mellitus (DM) [4]. Among the characteristics of MS, obesity—an excess of adipose tissue—has been shown to be an important indicator because it can be a source of proinflammatory cytokines, which may be linked to a greater risk for developing further insulin resistance as well as atherogenesis [5].

Ethnic disparities in the rates of CVD and DM have been noted. Previous studies in Canada and the UK indicated that people of South Asian origin had the highest rate of cardiovascular disease, people of European origin had an intermediate rate, and people of Chinese origin had the lowest rate [6–9]. In the U.S., a population-based telephone survey in California indicated that compared to Caucasians, even after adjusting for lifestyle and other risk factors, the likelihood of having type 2 diabetes was significantly higher for most other ethnic groups except Chinese and Vietnamese. Native Americans (OR: 3.8, 95% CI 2.0–7.4) were at the highest risk, and the risks of certain Asian Americans were even higher than those of African Americans (OR: 2.2, 95% CI 1.4–3.2), namely Filipino (OR: 4.0; 95% CI 2.2–7.1), Korean (OR: 3.5; 95% CI 2.0–6.0) and South Asian (OR: 3.3; 95% CI 1.9–5.7) [10].

The variations among ethnic groups can be partly attributed to the differences in their profiles of CMR, among which smoking, obesity, and metabolic syndrome are more prevalent among minority ethnicities [6, 11–16]. Indigenous peoples exhibited higher risk profiles [11, 13, 15, 16], and obesity was particularly worthy of note. In an international comparison study, among ten countries that had provided data on adult obesity, seven had higher rates (body mass index ≥ 30 kg/m^2^) in indigenous populations than in their benchmark populations. These countries included Australia (41.0% vs. 26.2%), Canada (37.8% vs. 22.6%), Greenland (23.1% vs. 16.1%), Myanmar (8.0% vs. 6.4%), New Zealand/Aotearoa (44.7% vs. 24.7%), Norway (32.3% vs. 26.1%), and the USA (39.0% vs. 19.2%) [16].

With heart disease, cerebrovascular disease and DM increasingly reported on the list of the top ten leading causes of death across high-income countries, efforts have been made to diminish health disparities in terms of the life expectancy across ethnic groups as advocated by the World Health Organization. Taiwan also faces the same public health challenge. The indigenous population in Taiwan has higher rates of heart disease, cerebrovascular disease and DM than the nonindigenous population. In 2017, their life expectancy at birth was estimated at 72.2 years, which was approximately 8.2 years less than that for the nonindigenous population, with a gap of 9.4 years for men and 7.1 for women [17].

Despite many studies portraying ethnic discrimination and disadvantaged social determinants of minority unhealthy behaviors and health care utilization to explain ethnic gaps [18–20], biological variations across different ethnic groups have also been noted [21, 22]. It has been proven that indigenous peoples in Taiwan originated in Southeast Asia, migrating to Taiwan approximately 11,000–26,000 years ago and later migrating to western and central Polynesia, and possibly to New Zealand. They have been demonstrated to be genetically related to Austronesians and are considered relatives of the indigenous people of Māori and the minority of Pacific descent in New Zealand [23, 24]. An investigation conducted in New Zealand by using ATP III criteria revealed that the rates of abdominal obesity and metabolic syndrome were much higher for ethnic minorities than for those of European descent in both men and women (in adults aged 40–59 years: abdominal obesity rate was 63.9% for Māori, 66.4% for Pacific islander and 36.9% for European descent in men; 75.2% for Māori, 91.8% for Pacific islander and 41.8% for European descent in women) [11].

A study analyzed data from a local adult health examination program for residents of Pingtung County located in the southern area of Taiwan in 2006 and found that the rates of abdominal obesity and metabolic syndrome in indigenous subjects were significantly higher than those in their Han counterparts (abdominal obesity: 83.3% > 56.4%; MS: 83.3% > 42.7%) [25], and the magnitudes of the disparity were relatively high (the ethnic gaps were 26.9% for abdominal obesity and 40.6% for MS). However, this study was limited due to its small size of 90 indigenous subjects. A later study analyzed data from Chiayi County’s health examination program for indigenous adults residing in the southwestern area of Taiwan in 2008, showing that the rates (abdominal obesity: 57.8%, metabolic syndrome: 42.6%) were not as high as those in the former; the significant risk factors for having metabolic syndrome were older age, lower education level, drinking alcohol, and chewing betel quid [26]. Recently, a community-based study conducted in the northern area of Taiwan in 2010–2013 also revealed similar results, in which the rate of metabolic syndrome in the indigenous population was 42.6% and associated risk factors for MS included older age and a lower educational level [27]. Unfortunately, these studies suffered from a lack of comparison information from the benchmark people, namely the ethnic Han. It is of interest to note that the rates of abdominal obesity and MS in indigenous population found in previous studies appeared to be approximately 10% or more higher than the rates in Han people found in the 2015–2017 Taipei health examination program (abdominal obesity was 40% and metabolic syndrome was 33%) [28]. The magnitude of ethnic disparity may vary by the socioeconomic levels of the living areas and by the distributions of age, sex and risk factors across ethnic groups.

Due to the existence of ethnic disparities in MS, obesity and other cardiometabolic risks, it is worth asking if ethnicity per se plays an important role, which may possibly be attributed to some biological reasons of different ethnic groups, or if ethnicity is only a manifestation of health behaviors shaped and varied by the socioeconomic levels of the living areas. To answer this question, studying people living in the same area to reduce the neighborhood effect may be a promising approach. Studies on ethnic disparities in metabolic syndrome and its risk factors in Taiwan are still rare; therefore, the objectives of this study were: (1) to detect the differences in the rates of metabolic syndrome, obesity and health behaviors between two ethnic groups (indigenous Tsou and nonindigenous Han) living in the same area and with similar age and sex distributions, and (2) to examine whether ethnicity per se plays a significant role in the occurrence of metabolic syndrome, while taking other risk factors including sociodemographic characteristics, obesity and health behaviors into consideration.

## Methods

In this study, we used data from a cross-sectional community survey that we conducted in cooperation with the Chiayi County Health Bureau—a local health government in southwestern Taiwan. The participants in the community survey were adult residents participating in Chiayi County’s Adult Health Examination Program (AHEP) during 2012–2013. This survey was approved by the Institutional Review Board of China Medical University Hospital (DMR101-IRB061), and signed informed consent was obtained from each participant. Face-to-face interviews were conducted using a structured questionnaire to collect information on sociodemographic attributes (including ethnicity, age, sex, marital status, educational levels and the employment), chronic diseases (including heart disease, stroke, hypertension, hyperlipidemia, diabetes, and gout), and health behaviors. Ethnicity obtained from participants’ self-reported ethnic identity was then classified based on the ethnic categories groups (Han—Chinese origins, indigenous peoples including 16 tribe peoples—Austronesian origins, and new immigrants) defined by the Taiwan government [29, 30]. The government officially recognized indigenous peoples based on the definition suggested by the United Nations [31] to distinct the pre-settlers (arrivals approximately 11,000–26,000 years ago) from the old immigrants of Han (arrivals since the seventeenth century) and the new immigrants (arrivals since the late twentieth century). Height and weight were assessed by using scales; waist circumference was assessed by using a tape measure taken around the abdomen at the level of the midpoint between the lower margin of the last palpable rib and the top of the iliac crest. Systolic and diastolic blood pressure were assessed by using a sphygmomanometer. Fasting (more than 8 h) blood specimens were collected to assess the concentrations of glucose, triglycerides, and cholesterol. The total number of adult residents participating in the community survey was 9067.

According to the definition published by the Health Promotion Administration, Ministry of Health and Welfare in Taiwan in 2007, metabolic syndrome refers to meeting three or more of the following five criteria: impaired fasting glucose (≥ 100 mg/dL) or taking hypoglycemic medication, abdominal obesity (waist circumference ≥ 90 cm in men and ≥ 80 cm in women), an elevated blood pressure (systolic ≥ 130 mmHg and/or diastolic ≥ 85 mmHg) or taking antihypertension medication, reduced HDL-C (< 40 mg/dL in men; < 50 mg/dL in women), and elevated triglycerides (≥ 150 mg/dL) or taking antihyperlipidemic medication. The above definition is for Asian people, which is modified from the NCEP-ATP III criteria [32]. In addition to waist circumference, body mass index (BMI) is also used to define obesity. For Asian people, a BMI greater than or equal to 27 kg/m^2^ is defined as obesity [33, 34].

Smoking, drinking and regular exercise behaviors were assessed by asking participants whether they smoked at least once a day, drank alcohol at least once a week, and engaged in regular exercise, where regular exercise was defined by asking whether the exercise was performed at least once a week, each session lasting for more than 30 min and engaging in this regular pattern of exercise for a persistent period of more than half a year. The self-reported responses were dichotomous (yes or no).

Information on healthy diet behaviors was obtained from self-reported intake within a week before the surveyed day. Participants were asked to recall their daily intake of vegetables and fruits (unit: servings/day) and weekly intake of fried food (unit: times/week) and soft drinks (unit: bottles/week). The disease status information was obtained by asking participants whether they had a chronic disease (including heart disease, stroke, hypertension, hyperlipidemia, diabetes, and gout) that was diagnosed by a physician before the survey day.

In this study, we excluded the invalid and missing information from the surveyed 9067 participants, and data from 8716 participants remained. Among the 8716 participants, only two ethnic groups were identified (667 indigenous Tsou and 8049 nonindigenous Han peoples). To better understand the ethnic disparities in the rates of MS and its associates without confounding from the age and sex distributions across the ethnic groups, a frequency matching strategy by age and sex with a ratio of 1 (Tsou) to 3 (Han) was used to select a comparable sample with equal distributions of age and sex between Tsou and Han from among the 8716 participants. Thus, 2668 participants (667 Tsou; 2001 matched Han) remained with a mean age of 56.9 years (SD: 14.5), and 22.0% were aged over 69 years.

**Fig. 1:**
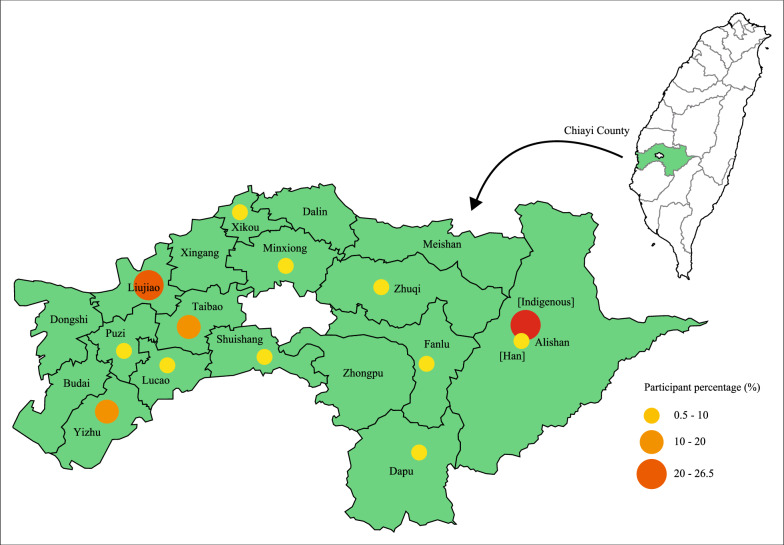
Geographic information on the percentage of indigenous Tsou and Han participants across Chiayi County’s Townships

According to the population statistics from the Council of Indigenous Peoples in Taiwan in June 2012, a total of 5599 aborigines reside in Chiayi County. Among them, Tsou tribe people comprised the majority (72%, n = 4026), and most of them reside (n = 3684) in the Alishan mountain area governed by Alishan Township [35]. In the present study, the 667 indigenous Tsou people all dwelled in Alishan Township. Figure 1 outlines the geographic information on the distribution of 667 indigenous Tsou and 2001 Han subjects across 18 Chiayi County’s Townships.

**Fig. 2:**
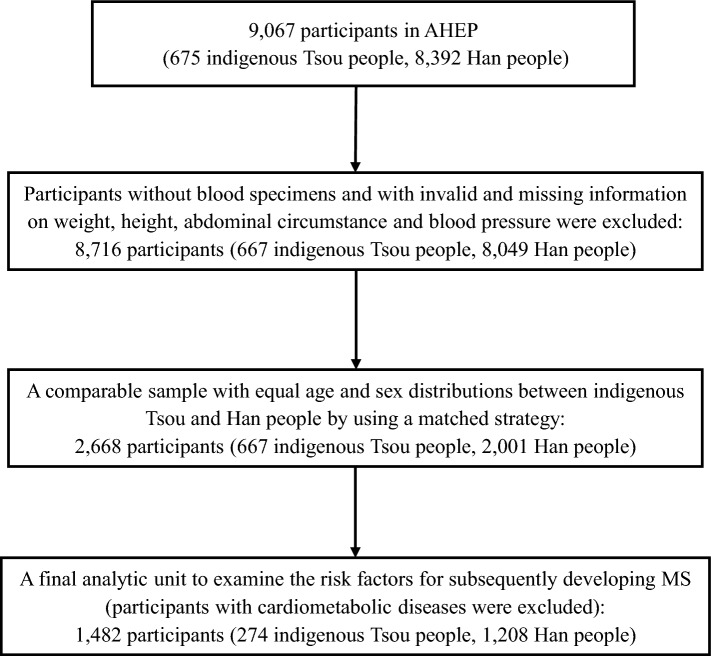
Flowchart of selecting the study subjects

Considering that participants with chronic diseases diagnosed before the surveyed day may have already modified their unhealthy behaviors due to disease treatments and this behavior modification could in turn bias the examined associations of health behaviors with the subsequent occurrence of metabolic syndrome, we excluded them from the analysis. Finally, an analytic unit of 1482 participants (274 indigenous Tsou and 1208 nonindigenous Han peoples) was obtained for further analyses to examine the ethnicity disparity in the likelihood of developing MS by controlling for other risk factors. The flowchart of selecting the analytic subjects is depicted in Fig. 2.

The statistical analyses in this study were conducted by using SPSS 25.0 for Windows. Chi-square tests, Student’s *t*-tests and simple logistic regression were applied for bivariate analyses, and multiple logistic regression models were applied for multivariate analyses.

## Results

In the matched sample, the rates of metabolic syndrome, abdominal obesity and obesity (based on Asian BMI criteria) in the indigenous Tsou people were significantly higher than those in Han people (54.0% > 29.1%, 72.4% > 41.5%, and 54.0% > 23.2%, respectively). The distributions of chronic diseases and health behaviors between the two ethnic groups were examined. All six diseases had significantly higher rates in Tsou people than in Han people (heart disease 14.7% > 7.4%, P < 0.001; stroke 2.8% > 1.3%, P = 0.007; hypertension 47.1% > 25.0%, P < 0.001; hyperlipidemia 13.3% > 10.2%, P = 0.024; diabetes 14.7% > 9.6%, P < 0.001; and gout 10.5% > 7.7%, P = 0.024). Regarding health behaviors, the rates of drinking alcohol and higher intake of fried food (≥ 3 times per week) were significantly higher in indigenous Tsou people than in Han people (17.5% > 13.6%, P = 0.014; and 6.4% > 4.4%, P = 0.034; respectively). In contrast, the rate of higher intake of soft drinks was significantly lower (10.6% < 13.8%, P = 0.034) in Tsou people than in Han people (Table 1).

**Table 1:** Comparisons between matched and final analytic samples and between Tsou and Han within each sample

Variables	The matched sample				The final analytic sample				P^c^ value
	Total N (%)	Tsou	Han	P value	Total N (%)	Tsou	Han	P value	
		(N = 667)	(N = 2001)			(N = 274)	(N = 1208)		
		N (%)	N (%)			N (%)	N (%)		
Sociodemographic characteristics									
Ethnicity									< 0.001
Han	2001 (75.0)				1208 (81.5)				
Tsou	667 (25.0)				274 (18.5)				
Sex				1.000				0.264	0.608
Male	1120 (42.0)	280 (42.0)	840 (42.0)		610 (41.2)	121 (44.2)	489 (40.5)		
Female	1548 (58.0)	387 (58.0)	1161 (58.0)		872 (58.8)	153 (55.8)	719 (59.5)		
Age				1.000				< 0.001	< 0.001
20–49 years	844 (31.6)	211 (31.6)	633 (31.6)		619 (41.8)	142 (51.8)	477 (39.5)		
50-59 years	692 (25.9)	173 (25.9)	519 (25.9)		397 (26.8)	68 (24.8)	329 (27.2)		
60-69 years	544 (20.4)	136 (20.4)	408 (20.4)		253 (17.1)	33 (12.0)	220 (18.2)		
≥ 70 years	588 (22.0)	147 (22.0)	441 (22.0)		213 (14.4)	31 (11.3)	182 (15.1)		
Mean ± SD	56.9 ± 14.5	56.9 ± 14.5	56.9 ± 14.5	0.986	53.0 ± 14.1	49.9 ± 13.6	53.6 ± 14.1	< 0.001	< 0.001
Education				< 0.001				< 0.001	0.028
Elementary school	880 (33.0)	161 (24.1)	719 (35.9)		469 (31.6)	62 (22.6)	407 (33.7)		
High school	1103 (41.3)	328 (49.1)	775 (38.7)		571 (38.5)	113 (41.2)	458 (37.9)		
At least with college	685 (25.7)	178 (26.7)	507 (25.3)		442 (29.8)	99 (36.1)	343 (28.4)		
Marital status				< 0.001				< 0.001	< 0.001
Single^a^	530 (19.9)	190 (28.5)	340 (17.0)		278 (18.8)	73 (26.6)	205 (17.0)		
Married	2138 (80.1)	477 (71.5)	1661 (83.0)		1204 (81.2)	201 (73.4)	1003 (83.0)		
Employed status				0.529				< 0.001	0.270
Yes	1176 (44.1)	287 (43.0)	889 (44.4)		627 (42.3)	89 (32.5)	538 (44.5)		
No	1492 (55.9)	380 (57.0)	1112 (55.6)		855 (57.7)	185 (67.5)	670 (55.5)		
With concurrent chronic disease^b^									
Heart disease	246 (9.2)	98 (14.7)	148 (7.4)	< 0.001					
Stroke	45 (1.7)	19 (2.8)	26 (1.3)	0.007					
Hypertension	814 (30.5)	314 (47.1)	500 (25.0)	< 0.001					
Hyperlipidemia	293 (11.0)	89 (13.3)	204 (10.2)	0.024					
Diabetes	290 (10.9)	98 (14.7)	192 (9.6)	< 0.001					
Gout	224 (8.4)	70 (10.5)	154 (7.7)	0.024					
Chronic disease, number				< 0.001					
0	1482 (55.5)	274 (41.1)	1208 (60.4)						
1–2	1016 (38.1)	317 (47.5)	699 (34.9)						
3–4	158 (5.9)	72 (10.8)	86 (4.3)						
≥ 5	12 (0.4)	4 (0.6)	8 (0.4)						
Health behavior									
Smoking				0.539				0.157	0.417
Yes	326 (12.2)	86 (12.9)	240 (12.0)		194 (13.1)	43 (15.7)	151 (12.5)		
No	2342 (87.8)	581 (87.1)	1761 (88.0)		1288 (86.9)	231 (84.3)	1057 (87.5)		
Drinking alcohol				0.014				0.003	0.876
Yes	390 (14.6)	117 (17.5)	273 (13.6)		214 (14.4)	55 (20.1)	159 (13.2)		
No	2278 (85.4)	550 (82.5)	1728 (86.4)		1268 (85.6)	219 (79.9)	1049 (86.8)		
Regular exercise				0.687				0.563	0.981
Yes	1254 (47.0)	309 (46.3)	945 (47.2)		786 (53.0)	141 (51.5)	645 (53.4)		
No	1414 (53.0)	358 (53.7)	1056 (52.8)		696 (47.0)	133 (48.5)	563 (46.6)		
Vegetable intake				0.432				0.085	0.516
< 3 servings/day	990 (37.1)	256 (38.4)	734 (36.7)		543 (36.6)	88 (32.1)	455 (37.7)		
≥ 3 servings/day	1678 (62.9)	411 (61.6)	1267 (63.3)		939 (63.4)	186 (67.9)	753 (62.3)		
Fruit intake				0.070				0.738	0.812
< 2 servings/day	1544 (57.9)	406 (60.9)	1138 (56.9)		852 (57.5)	160 (58.4)	692 (57.3)		
≥ 2 servings/day	1124 (42.1)	261 (39.1)	863 (43.1)		630 (42.5)	114 (41.6)	516 (42.7)		
Fried food intake				0.034				0.049	< 0.001
< 3 times/week	2537 (95.1)	624 (93.6)	1913 (95.6)		1404 (94.7)	253 (92.3)	1151 (95.3)		
≥ 3 times/week	131 (4.9)	43 (6.4)	88 (4.4)		78 (5.3)	21 (7.7)	57 (4.7)		
Soft drink intake				0.034				0.028	0.106
< 3 bottles/week	2320 (87.0)	596 (89.4)	1724 (86.2)		1262 (85.2)	245 (89.4)	1017 (84.2)		
≥ 3 bottles/week	348 (13.0)	71 (10.6)	277 (13.8)		220 (14.8)	29 (10.6)	191 (15.8)		
Unhealthy behavior, number				0.043				0.006	0.649
0	412 (15.4)	92 (13.8)	320 (16.0)		242 (16.3)	48 (17.5)	194 (16.1)		
1–2	1473 (55.2)	365 (54.7)	1108 (55.4)		794 (53.6)	138 (50.4)	656 (54.3)		
3–5	770 (28.9)	203 (30.4)	567 (28.3)		436 (29.4)	82 (29.9)	354 (29.3)		
≥ 6	13 (0.5)	7 (1.0)	6 (0.3)		10 (0.7)	6 (2.2)	4 (0.3)		
Obesity index									
Obesity (by BMI)				< 0.001				< 0.001	< 0.001
Yes	825 (30.9)	360 (54.0)	465 (23.2)		364 (24.6)	116 (42.3)	248 (20.5)		
No	1843 (69.1)	307 (46.0)	1536 (76.8)		1118 (75.4)	158 (57.7)	960 (79.5)		
Abdominal obesity				< 0.001				< 0.001	< 0.001
Yes	1313 (49.2)	483 (72.4)	830 (41.5)		592 (39.9)	163 (59.5)	418 (34.6)		
No	1355 (50.8)	184 (27.6)	1171 (58.5)		890 (60.1)	111 (40.5)	790 (65.4)		
Metabolic syndrome				< 0.001				< 0.001	< 0.001
Yes	943 (35.3)	360 (54.0)	583 (29.1)		372 (25.1)	123 (44.9)	249 (20.6)		
No	1725 (64.7)	307 (46.0)	1418 (70.9)		1110 (74.9)	151 (55.1)	959 (79.4)		

^a^Single includes unmarried, divorced, and widowed status

^b^Omits those without the disease

^c^Denotes P value obtained from the comparison between the matched and final analytic samples. Chi-square tests and Student’s *t*-tests were applied

By comparing the changes in the proportions of the studied attributes between the matched 2668 and the remaining 1482 samples, as shown in Table 1, we noted that among the excluded sample of 1186 participants, that is, among those individuals with any chronic disease diagnosed before the surveyed day, more were aboriginal, aged over 50 years, with lower educational levels, single, and employed; more were with the following three healthy behaviors: nonsmoking, lower intake of fried food and lower intake of soft drinks; and more were with the following four unhealthy behaviors: drinking alcohol, without regular exercise, lower intake of vegetables and lower intake of fruits. Despite the changes mentioned above, this exclusion did not cause most of the distributions of the studied attributes to differ significantly between the matched 2668 and the remaining 1482 samples, except for ethnicity (P < 0.001), age (P < 0.001), educational level (P = 0.028), marital status (P < 0.001) and intake of fried food (P < 0.001). However, a substantial number of the participants from the matched sample with obesity and MS were excluded because of the comorbid nature of their chronic diseases.

It is interesting to note that among those with metabolic syndrome (identified based on the definition including under treatment for previously diagnosed hypertension, hyperlipidemia, and/or diabetes; n = 943), up to 60.6% (571/943) were comorbid with other cardiometabolic diseases. The exclusion was over half of the 667 indigenous Tsou people (58.9% = 393/667) and over one-third of the 2001 Han participants (39.6% = 793/2001), which suggests that by comparing the rates of having any chronic diseases between both populations with equivalent age and sex distributions, the indigenous people’s health was in general worse.

The comparison result for the distributions of the sociodemographic characteristics, health behaviors, obesity and metabolic syndrome for those without any clinically diagnosed cardiometabolic diseases between the indigenous Tsou and nonindigenous Han groups showed that indigenous subjects were on average younger (49.9 ± 13.6 years) than their Han counterparts (53.6 ± 14.1 years). They were more likely to be single, with higher educational levels, and in an unemployed status. They had higher rates of drinking alcohol (20.1% > 13.2%, P = 0.003) and a higher intake of fried food (7.7% > 4.7%, P = 0.049), while their Han counterparts had a higher rate of consuming more soft drinks (15.8% > 10.6%, P = 0.028). Abdominal obesity (59.5% > 34.6%), BMI obesity (42.3% > 20.5%), and metabolic syndrome (44.9% > 20.6%) were significantly more common in the aborigines.

In Table 2, the results of crude prevalence odds ratios (PORs) for bivariate analyses showed that ethnicity, age, education levels, marital status, drinking alcohol, the number of unhealthy behaviors, and obesity were significantly associated with the occurrence of metabolic syndrome. Multiple logistic regression models were further applied to examine the adjusted PORs of the risk factors for MS, and the results are shown in Table 3. Model 1 only examined sociodemographic characteristics, and the results remained similar to the bivariate analyses except for the education levels and marital status. The final results are shown in Model 3, indicating that ethnic disparity significantly existed, in which the indigenous Tsou people were 2.62 times more likely to have MS than Han people (POR = 2.62, 95% CI 1.91–3.58, P < 0.001). The likelihood of having MS increased with age (compared to those aged 20–49 years, PORs for those aged 50–59 years, aged 60–69, and aged 70 years or over were 1.57, 2.17 and 3.25, respectively), and marriage could have a protective effect against MS (POR = 0.70, P = 0.023). Obesity was a salient risk factor, in which people with obesity were 5.26 times more likely to have MS than those without obesity (POR = 5.26, 95% CI 3.99–6.94, P < 0.001). Compared to zero unhealthy behaviors, having equal to or more than six unhealthy behaviors increased the chance for MS 6.71 times, as shown in Model 2b (POR = 6.17, 95% CI 1.43–26.73, P = 0.015). Nevertheless, this detrimental effect disappeared in Model 3, where obesity was taken into account. This change might suggest a possible indirect effect of unhealthy behavior on MS through its potential influence on obesity. In addition, the significant effect of drinking alcohol in the bivariate analysis disappeared when sociodemographic and other health behaviors were taken into consideration (see the results in Model 2a).

**Table 2:** The bivariate analyses of the relationships between the risk factors and metabolic syndrome (n = 1482)

Variables	Crude POR (95% CI)	P value
Sociodemographic characteristics		
Ethnicity (Reference: Han)	3.14 (2.38–4.13)	< 0.001
Sex (Reference: male)	0.98 (0.76–1.27)	
Age (Reference: 20–49 years)		
50–59 years	1.05 (0.83–1.33)	0.704
60–69 years	1.39 (1.03–1.87)	0.032
≥ 70 years	1.78 (1.31–2.42)	< 0.001
Education (Reference: elementary school)		
High school	1.32 (1.04–1.68)	0.022
At least with college	0.74 (0.57–0.97)	0.027
Marital status (Reference: single^a^)	0.70 (0.54–0.92)	0.009
Employed status (Reference: not employed)	1.13 (0.90–1.44)	0.296
Health Behavior		
Smoking (Reference: no)	1.29 (0.92–1.80)	0.141
Drinking alcohol (Reference: no)	1.40 (1.02–1.93)	0.037
Regular exercise (Reference: yes)	1.10 (0.87–1.39)	0.450
Vegetable intake (Reference: ≥ 3 servings/day)	0.84 (0.65–1.07)	0.160
Fruit intake (Reference: ≥ 2 servings/day)	1.08 (0.85–1.37)	0.534
Fried food intake (Reference: < 3 times/week)	1.26 (0.77–2.09)	0.360
Soft drink intake (Reference: < 3 bottles/week)	1.11 (0.80–1.54)	0.525
Unhealthy behavior, number (Reference: zero)		
1–2	1.30 (0.92–1.83)	0.145
3–5	1.26 (0.87–1.84)	0.224
≥ 6	8.74 (2.18–34.99)	0.002
Obesity	5.42 (4.18–7.03)	< 0.001

^a^Single includes unmarried, divorced, and widowed status. POR: denotes prevalence odds ratio. Simple logistic regression models were applied

**Table 3:** The multiple logistic regression models examining the risk factors for metabolic syndrome (n = 1482)

Variables	Model 1		Model 2a		Model 2b		Model 3	
	OR (95% CI)	P value	OR (95% CI)	P value	OR (95% CI)	P value	OR (95% CI)	P value
Sociodemographic characteristics								
Ethnicity (Reference: Han)	3.49 (2.61–4.68)	< 0.001	3.43 (2.56–4.61)	< 0.001	3.43 (2.56–4.60)	< 0.001	2.62 (1.91–3.58)	< 0.001
Sex (Reference: male)	0.98 (0.76–1.27)	0.891	0.99 (0.76–1.27)	0.907	0.99 (0.77–1.28)	0.924	0.97 (0.74–1.27)	0.823
Age (Reference: 20–49 years)								
50–59 years	1.43 (1.04–1.98)	0.03	1.42 (1.02–1.97)	0.035	1.41 (1.02–1.95)	0.040	1.57 (1.11–2.22)	0.011
60–69 years	2.04 (1.41–2.95)	< 0.001	2.06 (1.42–2.99)	< 0.001	2.04 (1.41–2.95)	< 0.001	2.17 (1.46–3.23)	< 0.001
≥ 70 years	2.40 (1.63–3.53)	< 0.001	2.39 (1.63–3.52)	< .001	2.39 (1.62–3.52)	< 0.001	3.25 (2.15–4.91)	< 0.001
Education (Reference: elementary school)								
High school	1.05 (0.77–1.42)	0.766	1.04 (0.77–1.41)	0.791	1.05 (0.78–1.42)	0.747	1.04 (0.75–1.43)	0.828
At least with college	0.90 (0.64–1.27)	0.541	0.88 (0.63–1.25)	0.486	0.90 (0.64–1.27)	0.550	1.01 (0.70–1.46)	0.951
Marital status (Reference: single^a^)	0.80 (0.60–1.06)	0.115	0.78 (0.59–1.04)	0.090	0.79 (0.59–1.05)	0.097	0.70 (0.52–0.95)	0.023
Employed status (Reference: not employed)	1.10 (0.85–1.42)	0.484	1.07 (0.82–1.39)	0.614	1.09 (0.84–1.42)	0.520	1.02 (0.78–1.35)	0.865
Health Behavior								
Smoking (Reference: no)			1.02 (0.70–1.50)	0.912				
Drinking alcohol (Reference: no)			1.24 (0.87–1.79)	0.238				
Regular exercise (Reference: yes)			1.07 (0.84–1.38)	0.576				
Vegetable intake (Reference: ≥ 3 servings/day)			0.84 (0.64–1.11)	0.219				
Fruit intake (Reference: ≥ 2 servings/day)			1.07 (0.82–1.39)	0.639				
Fried food intake (Reference: < 3 times/week)			1.01 (0.59–1.75)	0.967				
Soft drink intake (Reference: < 3 bottles/week)			1.21 (0.85–1.72)	0.301				
Unhealthy behaviors, number (Reference: zero)								
1–2					1.37 (0.95–1.96)	0.089	1.28 (0.88–1.88)	0.197
3–5					1.27 (0.86–1.88)	0.226	1.16 (0.77–1.75)	0.480
≥ 6					6.17 (1.43–26.73)	0.015	4.11 (0.92–18.47)	0.065
Obesity							5.26 (3.99–6.94)	< 0.001
Model’s information								
− 2 log Likelihood	1567.43		1562.08		1559.29		1417.91	
Likelihood ratio statistic (G2)	102.62	< 0.001	107.97	< 0.001	110.76	< 0.001	252.14	< 0.001

^a^Single included unmarried, divorced, and widowed status

To our knowledge, obesity has been previously demonstrated to be an important predictor for developing metabolic syndrome and cardiovascular diseases. As Table 1 shows, there were significantly higher rates of having more unhealthy behaviors and obesity in indigenous Tsou people than in nonindigenous Han people. An examination of the net effect of ethnicity on obesity by controlling for the number of unhealthy behaviors was further implemented, and the result is shown in Table 4. In these results, compared to zero unhealthy behaviors, having equal to or more than six unhealthy behaviors could increase the risk for obesity by almost fourfold (POR = 3.98, 95% CI 1.02–15.51, P = 0.047). Nevertheless, being indigenous Tsou remained a significant risk factor (POR = 3.00, 95% CI 2.24–4.00). This result suggests that ethnicity per se plays a vital role in the risk of being obese.

**Table 4:** The multiple logistic regression models of the risk factors for obesity (n = 1482)

Variables	Model 1		Model 2	
	POR (95% CI)	P value	POR (95% CI)	P value
Sociodemographic characteristics				
Ethnicity (Reference: Han)	3.05 (2.28–4.07)	< 0.001	3.00 (2.24–4.00)	< 0.001
Sex (Reference: male)	0.98 (0.76–1.26)	0.853	0.98 (0.76–1.26)	0.852
Age (Reference: 20–49 years)				
50–59 years	0.85 (0.62–1.16)	0.306	0.83 (0.61–1.14)	0.257
60–69 years	1.10 (0.77–1.57)	0.618	1.09 (0.76–1.56)	0.641
≥ 70 years	0.57 (0.37–0.87)	0.009	0.56 (0.36–0.85)	0.007
Education (Reference: elementary school)				
High school	1.10 (0.81–1.49)	0.556	1.10 (0.81–1.49)	0.544
At least with college	0.73 (0.52–1.02)	0.064	0.73 (0.52–1.02)	0.062
Marital status (Reference: single^a^)	1.22 (0.91–1.65)	0.187	1.21 (0.90–1.63)	0.209
Employed status (Reference: not employed)	1.19 (0.92–1.55)	0.185	1.18 (0.91–1.54)	0.206
Unhealthy behaviors, number (Reference: zero)				
1–2			1.28 (0.89–1.83)	0.179
3–5			1.37 (0.93–2.01)	0.114
≥ 6			3.98 (1.02–15.51)	0.047
Model’s information				
− 2 log Likelihood	1581.23		1575.6	
Likelihood ratio statistic (G^2^)	71.1	< 0.001	76.72	< 0.001

^a^Single included unmarried, divorced, and widowed status

**Fig. 3:**
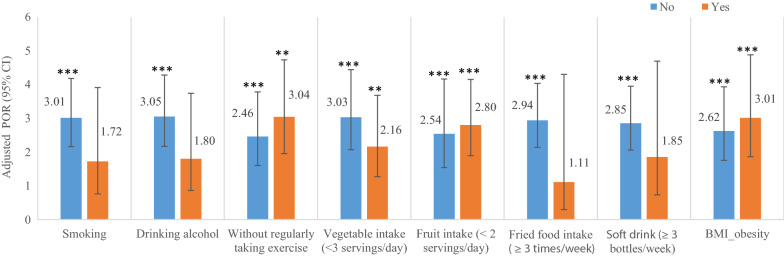
The adjusted prevalence odds ratio of aborigines for MS—by health behavior and by obesity. *0.01 ≤ P < 0.05, **0.001 ≤ P < 0.01, ***P < 0.001

Furthermore, we stratified the final analytic sample by the same behaviors to examine the association of simply being indigenous people with the occurrence of MS and found that even for those engaging in the same healthy behavior, the indigenous people (Tsou) were still more likely to have MS than Han people (adjusted PORs ranging 2.46–3.05, all corresponding P values < 0.001). For those exhibiting the same unhealthy behaviors, the indigenous people also had significantly higher likelihood than Han people (adjusted PORs ranging 2.16–3.04, all corresponding P values < 0.01) except for smoking, drinking alcohol, a higher intake of fried food and a higher intake of soft drinks (adjusted PORs ranging 1.11–1.85, all corresponding P values > 0.05) (Fig. 3). For people who had any one of the above four unhealthy behaviors, there was no difference in the risk of developing MS between the two ethnicities, suggesting the importance of engaging in healthy behaviors to prevent MS.

However, it is hard to ignore the net effect of being aborigines, as even when engaging in the same healthy behavior, simply being the indigenous people of Tsou did have a significantly greater likelihood of having MS. In addition, while conducting stratified analyses by the subgroups of without and with obesity (n = 1118 and n = 364, respectively), we still found an existence in the ethnic disparity in the likelihood of having MS (adjusted POR = 2.62, 95% CI 1.75–3.93 and adjusted POR = 3.01, 95% CI 1.86–4.88, respectively; see Fig. 3).

## Discussion

Obesity has been demonstrated to be the major risk factor for individuals to have MS, and this is particularly salient for the indigenous people of Tsou in the present study. The significantly higher rate of obesity in Tsou people (42.3%) than in Han people (20.5%) could partly explain the difference in the rates of MS between the indigenous (44.9%) and Han people (20.6%).

It generally assumed that unhealthy behaviors such as smoking, drinking alcohol, not regularly exercising, having low vegetable and fruit intake, and consuming more fried foods and soft drinks are mainly shaped and habituated by social determinants such as the culture, marital status, individual socioeconomic status (e.g., educational levels, employment status), all of which could directly or indirectly affect the likelihood of having MS. However, in the present study, unhealthy behaviors did not have a direct influence on the likelihood of having MS. Although drinking alcohol was found to be more prevalent among the indigenous people of Tsou than in Hans (20.1% > 13.2%, P = 0.003) and was significantly associated with MS in the bivariate analyses, its influential effect disappeared in the multivariate analyses after taking other risk factors into consideration.

It has been proposed that Taiwanese indigenous peoples have in their biological roots a greater risk of obesity [36], which may in turn increase their risk of having MS. In the analyses of the 1482 subjects, being indigenous Tsou people had a three times greater likelihood of having obesity than being nonindigenous Han people (adjusted POR = 3.00, 95% CI 2.24–4.00, P < 0.001). This brings up an important issue about preventing indigenous peoples from having MS, which is that the implementation of appropriate weight control programs and the enhancement of practicing healthy behaviors should be considered a top priority.

One strength of the present study is that we compared two ethnic groups with equal distributions of age and sex and demonstrated the existence of ethnic differences in the rates of metabolic syndrome, obesity, and their practices of healthy behaviors. Another strength is the exclusion of the sample with hypertension, hyperlipidemia, diabetes and other comorbidities to examine the risk factors for developing symptoms of metabolic syndrome in the future. In this way, the analytic results could disentangle the relationships between the possible predictors and the occurrence of the symptoms, although this exclusion did make the indigenous group on average younger than their Han counterparts.

The present study suffered from a small sample size of the indigenous people, resulting in a limitation of the generalization of the analytic results. However, we propose possible biological factors of aborigines are associated with their higher risks for obesity and metabolic syndrome. An interesting study in New Zealand demonstrated that the biological profiles associated with impaired metabolic homeostasis, such as gut hormones, pancreatic hormones and proinflammatory cytokines, appeared to differ between Māori and non-Māori individuals, independent of the presence of obesity, diabetes and other covariates [37], which potentially provided an explanation for the increased propensity to develop obesity and diabetes in the Māori population. On the basis of the existing findings that the ancestors of aboriginal Taiwanese and Māori people have the same origin [38], the rooted biological factors in Taiwanese indigenous peoples could potentially lead them to be more vulnerable to having MS than Han people.

In addition, risk genes were noted, such as UCP and LEP genes. Evidence from Taiwanese aboriginal studies showed that the V-A-T haplotype within the UCP2-UCP3 gene cluster increased the aboriginal’s risk for obesity [39], the frequency distribution of the risky allele of UCP2 (A-3751G) between indigenous and Han peoples was significantly different [40], and a genetically recessive role of the LEP −2548 G/G homozygote in developing extreme obesity for aborigines was suggested [36]. Despite these studies suffering weaknesses of insufficient sizes of the study subjects and a limited number of alleles of the candidate genes, possible differences in genetic factors did matter [41] and have the potential to explain the ethnic disparity in metabolic syndrome, which deserves further exploration to generate more effective interventions.

## Conclusions

By comparing two ethnic adult populations residing in the same area and with similar age and sex distributions, our study demonstrated the existence of ethnic disparities in the occurrences of metabolic syndrome, obesity problems, and unhealthy behaviors of drinking alcohol and a greater intake of fried food for which the indigenous Tsou people had significantly higher rates than their nonindigenous Han counterparts. Simply being indigenous Tsou significantly increased their risk for having MS and for being obese shown in the study results of examining the associated risk factors demonstrated that ethnicity per se did play a significant role in the occurrence of MS, suggesting possible biological reasons rooted in their origins that need further exploration. In addition, obesity was singled out as the number one culprit for the risk of having MS, and the cumulative appearances of unhealthy behaviors could predict the occurrence of being obese, which in turn would potentially increase the risk of developing metabolic syndrome.

As MS and obesity was much more prevalent in the indigenous Tsou people, and the cumulative number of unhealthy behaviors was also greater among them, community intervention programs to decrease the occurrence of metabolic syndrome need to make more efforts toward behavioral changes and weight control strategies with better cultural sensitivity to diminish ethnic health disparities.

## Data Availability

The datasets used during the current study are available from the corresponding author on reasonable request.

## Abbreviations

AHEP: Adult health examination program
MS: Metabolic syndrome
CVDs: Cardiovascular diseases
DM: Diabetes mellitus
NCEP-ATP III: National Cholesterol Education Programme Adult Treatment Panel III
CMR: Cardiometabolic risk
BMI: Body mass index
